# Knockout of the Chlorophyll a Oxygenase Gene *OsCAO1* Reduces Chilling Tolerance in Rice Seedlings

**DOI:** 10.3390/genes15060721

**Published:** 2024-06-02

**Authors:** Jiayi Xiong, Genping Wen, Jin Song, Xiaoyi Liu, Qiuhong Chen, Guilian Zhang, Yunhua Xiao, Xiong Liu, Huabing Deng, Wenbang Tang, Feng Wang, Xuedan Lu

**Affiliations:** 1 College of Agronomy, Hunan Agricultural University, Changsha 410128, China; xjiayi@stu.hunau.edu.cn (J.X.); 18627419269@163.com (G.W.); songjin1225@126.com (J.S.); 18473859293@163.com (X.L.); cqh924@163.com (Q.C.); zhang_gl604@hunau.edu.cn (G.Z.); yhxiao@hunau.edu.cn (Y.X.); xiongliu@whu.edu.cn (X.L.); denghuabing@hunau.edu.cn (H.D.); wangfennghifi@hunau.edu.cn (F.W.); 2 Yuelushan Laboratory, Changsha 410128, China; tangwenbang@hhrrc.ac.cn; 3 State Key Laboratory of Hybrid Rice, Hunan Hybrid Rice Research Center, Hunan Academy of Agricultural Sciences, Changsha 410125, China

**Keywords:** rice, chilling stress, chlorophyllide *a* oxygenase, yield

## Abstract

Chilling stress is one of the main abiotic factors affecting rice growth and yield. In rice, chlorophyllide *a* oxygenase encoded by *OsCAO1* is responsible for converting chlorophyllide *a* to chlorophyllide *b*, playing a crucial role in photosynthesis and thus rice growth. However, little is known about the function of *OsCAO1* in chilling stress responses. The presence of the *cis*-acting element involved in low-temperature responsiveness (LTR) in the *OsCAO1* promoter implied that *OsCAO1* probably is a cold-responsive gene. The gene expression level of *OsCAO1* was usually inhibited by low temperatures during the day and promoted by low temperatures at night. The *OsCAO1* knockout mutants generated by the CRISPR-Cas9 technology in rice (*Oryza sativa* L.) exhibited significantly weakened chilling tolerance at the seedling stage. *OsCAO1* dysfunction led to the accumulation of reactive oxygen species and malondialdehyde, an increase in relative electrolyte leakage, and a reduction in antioxidant gene expression under chilling stress. In addition, the functional deficiency of *OsCAO1* resulted in more severe damage to chloroplast morphology, such as abnormal grana thylakoid stacking, caused by low temperatures. Moreover, the rice yield was reduced in *OsCAO1* knockout mutants. Therefore, the elevated expression of *OsCAO1* probably has the potential to increase both rice yield and chilling tolerance simultaneously, providing a strategy to cultivate chilling-tolerant rice varieties with high yields.

## 1. Introduction

In nonvascular and vascular plants, such as green algae and prochlorophytes, chlorophyll (Chl) plays a central role in photosynthesis by harvesting light energy, transferring excitation energy to reaction centers, and driving charge separation reactions in reaction centers [1]. Five types of chlorophylls from natural photosynthetic organisms have been characterized to date: Chl *a*, Chl *b*, Chl *c*, Chl *d*, and Chl *f* [2,3]. Chl *a*, the most abundant Chl pigment, is present in the photosynthetic reaction centers and the light-harvesting antennae of almost all oxygenic photosynthetic organisms, including plants [4]. Chl *b*, the second-most-abundant Chl, functions only as the accessory Chl in the light-harvesting system of plants to bring photons to the reaction centers [5]. Chl *a* and Chl *b* have identical structures, except for the side chain at C-7, which is a methyl group in Chl *a* and a formyl group in Chl *b* [6]. Chl *b* binds and stabilizes many of the light-harvesting complex (LHC) proteins [7]. The antenna size contributing to the efficiency of photosynthesis was enlarged by the over-biosynthesis of Chl *b* [8]. Therefore, the biosynthesis of Chl *b* is important for the improvement of photosynthetic efficiency and thus crop productivity.

In *Arabidopsis*, the translation products of a nuclear gene *AtCAO* (chlorophyll *a* oxygenase) are imported into the chloroplast [9]. In chloroplasts, *AtCAO* catalyzes two-step monooxygenase reactions and converts Chl *a* to Chl *b* at the chloroplast’s inner membrane [5,9,10,11]. CAO is widespread in higher plants, such as prochlorophytes and chlorophytes [10,11,12]. The CAO protein in higher plants consists of four parts: a transit peptide and A, B, and C domains. The A domain in the N terminus of CAO confers protein instability and thus prevents the excess accumulation of Chl *b* and photodamage during the greening of *Arabidopsis* seedlings [13,14,15]. The C domain contains a Rieske cluster and an iron-binding motif and has catalytic functions [12,16]. The overexpression of B together with C domains led to a drastically decreased Chl *a*/*b* ratio and a significant delay of both developmental and dark-induced leaf senescence [17]. The controlled up-regulation of Chl *b* biosynthesis through the overexpression of *AtCAO* in tobacco increased both the antenna size and the electron transport rates, leading to enhanced carbon dioxide assimilation and starch and dry matter accumulation [18].

In the rice genome, two genes (*OsCAO1* and *OsCAO2*) are highly homologous to *AtCAO*. The *OsCAO1* knockout mutant lines exhibited pale green leaves, indicating Chl *b* deficiency, while the knockout mutations of *OsCAO2* did not change leaf color [19]. Furthermore, *OsCAO1* expression was induced by light and preferentially expressed in photosynthetic tissues, whereas *OsCAO2* was repressed by light exposure and increased under dark conditions [19]. It was suggested that *OsCAO1* plays a major role in Chl *b* biosynthesis, and *OsCAO2* may function in darkness [19]. A rice mutant, *pale green leaf* (*pgl*), harbored a single-base substitution in the coding region of *OsCAO1*, which resulted in a premature translational termination [20]. Almost no Chl *b* could be detected in the *pgl* mutant [20]. The *pgl* mutant showed a lower Chl content with a reduced and disorderly thylakoid ultrastructure, which decreased the photosynthesis rate and resulted in reduced grain yield and quality [20]. Moreover, *pgl* exhibited a more serious senescence phenotype than the WT under both natural and dark-induced conditions with more severe reactive oxygen species accumulation [20]. In addition, high temperatures accelerated *pgl* aging, indicating that *OsCAO1* or Chl *b* plays important roles in resisting high-temperature stress [20]. Another allele of *OsCAO1* with a two-base pair deletion in the ninth exon in Zhefu 802 (ZF802, an *indica* rice variety) also played an important role in Chl *b* content and thus leaf color regulation [21]. *OsCAO1* overexpression increased Chl *b* synthesis, accompanied by a higher photosynthetic rate and heavier 1000 grain weight than wild type ZF802 [21]. In addition, the knockout of *OsCAO1* influenced the grain quality characteristics of Dongjin (a *japonica* rice variety) [22].

Rice is a crucial crop for feeding a large part of the global population. Rice plants, especially the *indica* rice sub-population, are susceptible to low-temperature stress, leading to great yield loss under cold stress [23]. Rice photosynthesis is sensitive to low temperatures, and the photosynthetic rate is significantly reduced under chilling stress [24,25]. Chilling stress causes damage to photosynthetic machinery and activity and chloroplast development [26,27]. Chloroplasts are capable of detecting chilling and are the important plant cell organelles strongly influenced by chilling in plants [28]. Cold temperatures cause the photo-membranes to become rigid and slow down enzyme activity due to alterations in their constitution and formation [29]. However, the effects of *CAO* genes on low-temperature responses and chilling tolerance regulation remain elusive.

In this study, it was found that the promoter of *OsCAO1* harbors multiple stress-responsive motifs, such as LTR. *OsCAO1* gene expression responds to chilling stress. The knockout mutations of *OsCAO1* in the background of 9311 (an elite *indica* cultivar, widely used as the male parent of two-line hybrid rice in China) resulted in dramatically decreased survival rates of seedlings, the over-accumulation of ROS and MDA, increased electronic leakage, reduced antioxidant enzyme activities, impaired chloroplast morphology and Chl abnormal metabolism, and decreased yield of rice plants. Moreover, the functional disruption of *OsCAO1* affected the expression of antioxidant genes and cold-related transcription factor encoding genes.

## 2. Results

### 2.1. Analysis of Cis-Acting Elements of the OsCAO1 Promoter

**Figure 1:**
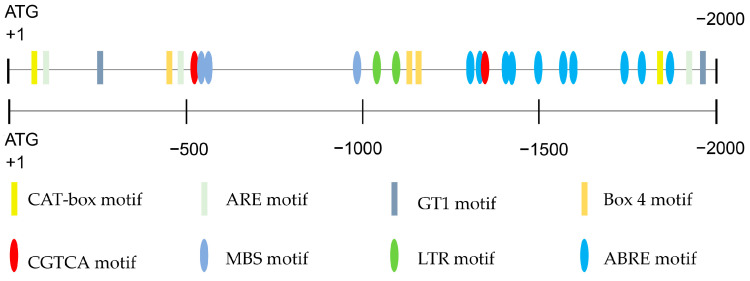
Promoter sequences (2.0 kb) of *OsCAO1* were analyzed by PlantCARE. Different colored and shaped boxes stand for different *cis*-elements. CAT box motif: *cis*-acting regulatory element related to meristem expression. ARE motif: *cis*-acting regulatory element essential for anaerobic induction. GT1 motif: light-responsive element. Box 4 motif: part of a conserved DNA module involved in light responsiveness. CGTCA motif: motif involved in MeJA responsiveness. MBS motif: MYB binding site involved in drought inducibility. LTR motif: motif involved in low-temperature responsiveness. ABRE motif: motif involved in the abscisic acid responsiveness.

The *cis*-acting element motifs in the promoter region control gene regulation at the transcriptional level [30]. To investigate whether the expression of the *OsCAO1* gene may respond to low temperatures, the promoter sequence of *OsCAO1* was analyzed by PlantCARE (a plant *cis-acting* regulatory element database) [31]. The results demonstrated that the promoter region of the *OsCAO1* gene contains a series of *cis*-elements related to stress resistance and hormone signaling, such as the ABRE motif (abscisic acid regulatory element, ACGTG), CGTCA motif (MeJA responsiveness element, CGTCA), CAT box motif (meristem expression element, GCCACT), ARE motif (anaerobic induction element, AAACCA), MBS motif (drought-induced element, CAACTG), GT1 motif (light responsive element, GGTTAA), Box 4 motif (part of a conserved DNA module involved in light responsiveness, ATTAAT), and LTR motif (low-temperature-responsive element, CCGAAA) (Figure 1). It was indicated that *OsCAO1* gene expression may respond to low temperatures through the LTR motif.

### 2.2. Gene Expression of OsCAO1 Responds to Low-Temperature Stress at the Seedling Stage

**Figure 2:**
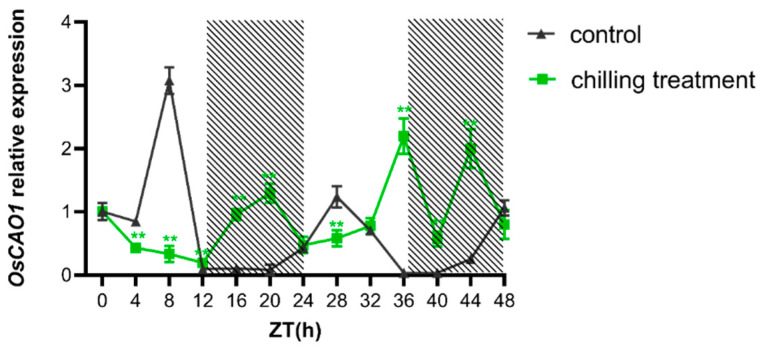
Effects of chilling temperature at different ZTs on the expression of *OsCAO1*. Rice seedlings were grown under a photoperiod of 12 h light/12 h dark at normal temperature (28 °C) until the trefoil stage when they were transferred to low temperatures (12 °C) for 48 h. Starting from ZT0, samples were taken every 4 h. Controls were seedlings sampled at the same time point while still growing at a normal temperature. Gene expression levels were normalized to *OsActin* and presented as values relative to that of control seedlings at ZT0. Significance analysis of gene expression at the same time point of low temperatures and normal temperatures was performed. Error bars indicate ± SD (*n* = 3). ** *p* < 0.01 (Student’s *t*-test).

To further investigate whether and how *OsCAO1* gene expression responds to low temperatures, we compared the RNA levels of *OsCAO1* in seedlings grown under normal temperatures with those in seedlings grown under chilling conditions for the same time during a diurnal light/dark cycle. As shown in Figure 2, chilling treatment (12 °C) increased *OsCAO1* gene expression by 103%, 924%, 1565%, 6135%, 1706%, and 782% at night or dusk ZT12 (Zeitgeber time), ZT16, ZT20, ZT36, ZT40, and ZT44, respectively, compared to room temperature. However, the chilling treatment applied near dawn and during daytime, including ZT4, ZT8, and ZT28, decreased the *OsCAO1* RNA level to 49.11%, 89.10%, and 53.14% of the control, respectively (Figure 2). It was suggested that the *OsCAO1* is a cold-responsive gene, and the circadian clock interacts with low temperatures to regulate *OsCAO1* gene expression.

### 2.3. Knockout of OsCAO1 Decreased the Chilling Tolerance in Rice

**Figure 3:**
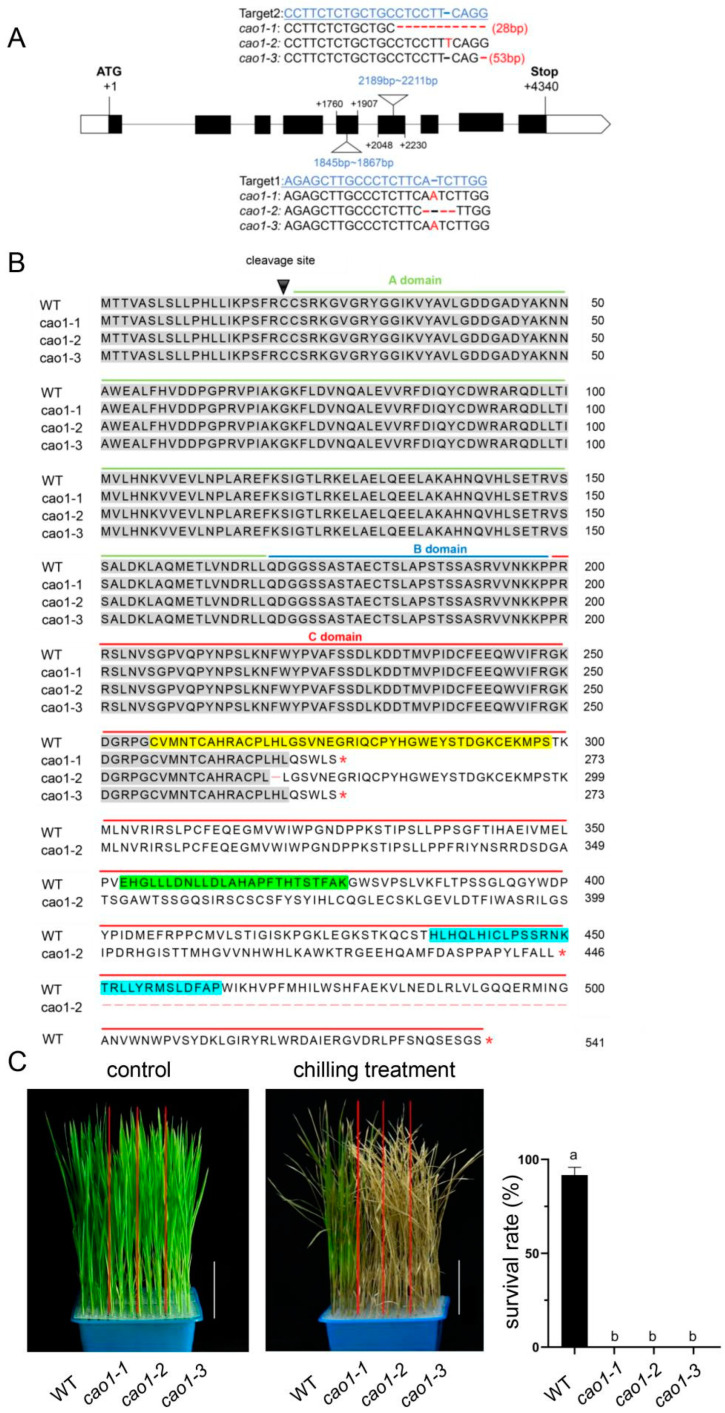
*OsCAO1* is essential for the chilling tolerance of rice seedlings. (**A**) Schematics of the two CRISPR/Cas9 target sites and the mutation sequences. The 5th and 6th exons of *OsCAO1* were targeted by CRISPR/Cas9 using two single-guide RNAs (Target 1 and Target 2). The exons, introns, and 5′- and 3′-UTRs of the gene are indicated by black rectangular boxes, black lines, and white rectangular boxes, respectively. The DNA sequences near the gene editing site in the genetic material are shown above and below the gene structure diagram. Missing bases in the mutant compared to the WT are indicated by short red lines and inserted bases are highlighted in red font; the total length of the missing bases is shown in the brackets. (**B**) The OsCAO1 protein in the WT was compared to three mutated proteins in *cao1-1*, *cao1-2*, and *cao1-3* mutants. The triangle indicates the transit peptide cleavage site in rice. The amino acids in the A, B, and C domains were marked with a green, blue, and red line, respectively. Amino acids identical to the WT sequence were highlighted in dark gray. The red “*“represents the termination of the translation. The Rieske FeS site, the mononuclear Fe site, and chlorophyllide a oxygenase unique conserved site were highlighted in yellow, green, and azure blue, respectively. (**C**) Control: the seedlings of the WT and *OsCAO1* knockout mutants grown continuously under normal temperature. Chilling treatment: seedlings that have undergone 5 days of low-temperature treatment (12 °C) and then recovered for 7 days. The rightmost bar chart represents the survival rate of seedlings after chilling treatment and recovery. Bar = 5 cm. Data are means ± SD. Different letters indicate significant differences (*p* < 0.05, Student’s *t*-test).

To reveal the role of *OsCAO1* in the regulation of chilling tolerance in *indica* rice, three independent knockout lines, *cao1-1*, *cao1-2*, and *cao1-3*, were generated through CRIPSR-Cas9 technology in the background of 9311, a well-known male parent of hybrid *indica* rice (Figure 3A). The mutation in *cao1-1* caused 1 bp insertion at +1861 bp and 28 bp deletion from +2201 bp, while the *cao1-3* mutant contains the same 1 bp insertion at +1861 bp and 53 bp deletion from +2210 bp (Figure 3A). Both mutations in *cao1-1* and *cao1-3* caused premature stop codons and thus truncated proteins (273 amino acids in *cao1-1* and *cao1-3*; 541 amino acids in the WT) (Figure 3B). For the *cao1-2* mutant, there is a 3 bp deletion from 1861 bp to 1863 bp and a 1 bp insertion at 2207 bp, resulting in a frameshift mutation. The corresponding protein in the *cao1-2* mutant lacked histidine at position 269, and the residues from the 338th amino acid were completely different from WT-OsCAO1 (Figure 3A,B). The truncated proteins in *cao1-1* and *cao1-3* mutants still retain the A domain, B domain, and about 20% of the length of the C domain of the WT-OsCAO1 protein, while the mutated protein in *cao1-2* retains A, B, and about 40% of the length of the C domain (Figure 3B). After 5 d of chilling treatment, all the mutant seedlings wilted and died, whereas the survival rate of the WT seedlings was about 91.66% (Figure 3C). These results demonstrated that *OsCAO1* plays a positive role in the regulation of rice chilling tolerance, and its intact C domain is crucial for this role.

### 2.4. OsCAO1 Dysfunction Leads to the Increase in ROS, MDA, and Relative Electrolyte Leakage under Low-Temperature Stress

**Figure 4:**
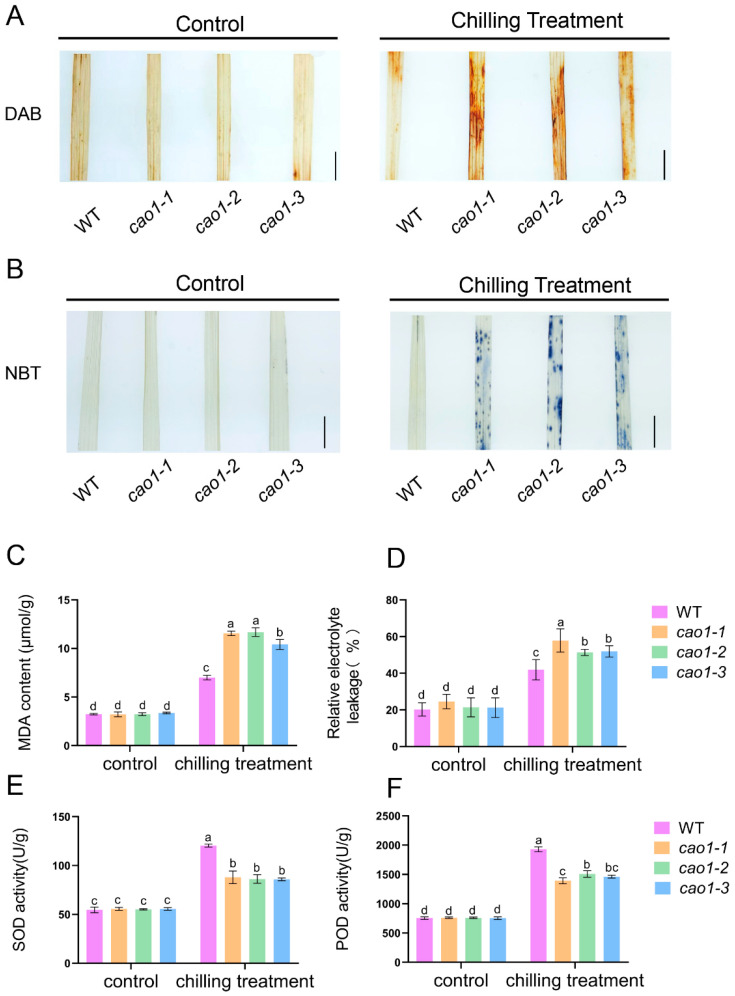
*OsCAO1* affected ROS and MDA accumulation, electrolyte leakage, and antioxidant enzyme activities under chilling stress. (**A**) DAB staining to show H_2_O_2_ accumulation. (**B**) NBT staining to show O_2_^.−^ accumulation. Bar = 0.5 cm. (**C**) Quantitative measurement of MDA content. (**D**) Relative EL. (**E**) SOD activity. (**F**) POD activity in the WT and *OsCAO1* knockout mutants. Control: normal temperature. Data are means ± SD (*n* = 3). Different letters indicate significant differences among plant materials by two-way analysis of variance (temperature and plant materials) (*p* < 0.05).

ROS (reactive oxygen species) over-accumulate in plants after low-temperature stress treatment, which, in turn, produces excessive amounts of toxic compounds, such as malondialdehyde (MDA), and disrupts cell membranes [32]. To explore whether *OsCAO1* regulated chilling tolerance through changing ROS homeostasis, the production of two kinds of ROS, H_2_O_2_, and O_2_^−^, were investigated through DAB and NBT staining, respectively. Chilling treatment resulted in increased intensity of leaf staining of all the plant materials. Although there was no significant difference in the staining intensity of the mutant leaves compared with the WT leaves under normal temperatures (control), the intensities of both staining were obviously higher in chilling-treated *OsCAO1* mutants than in chilling-treated WT (Figure 4A,B). Furthermore, under chilling treatment, the harmful MDA in *cao1-1*, *cao1-2,* and *cao1-3* mutants was 164.85%, 166.61%, and 148.58% relative to the WT, respectively (Figure 4C). Relative electrolyte leakage (EL) is the marker of cell membrane injury caused by abiotic stress [33]. As expected, chilling stress caused an increase in the relative EL of all mutant lines and the WT. However, the relative EL in *cao1-1*, *cao1-2*, and *cao1-3* mutants was 137.92%, 122.52%, and 123.84% relative to the WT, respectively, under the cold condition (Figure 4D). It was suggested that *OsCAO1* may reduce the accumulation of ROS and MDA and alleviate cell membrane damage under chilling stress.

The antioxidant enzymes SOD (superoxide dismutase) and POD (peroxidase) scavenge excess ROS, thereby protecting cells from stress [34]. To investigate the role of *OsCAO1* in scavenging ROS, we assayed the enzymatic activities of SOD and POD. Before cold treatment, there was no significant difference in the activities of the two antioxidant enzymes between the WT and *OsCAO1* knockout mutants. After low-temperature treatment, the SOD enzyme activities of the *cao1-1* mutant increased from 54.71% to 58.31%, whereas the WT increased dramatically by 120.46% (Figure 4E). Similarly, the *OsCAO1* mutant showed a significantly lower increase in POD activity under cold stress compared to the WT (Figure 4F). These results suggested that the disruption of *OsCAO1* may inhibit the intracellular antioxidant enzyme activities in response to the cold stress stimulus, thus negatively regulating the ROS scavenging and self-protection processes in rice.

### 2.5. OsCAO1 Affected the Changes of Chlorophyll Levels in Normal Temperature and Low-Temperature Rice Seedlings

**Figure 5:**
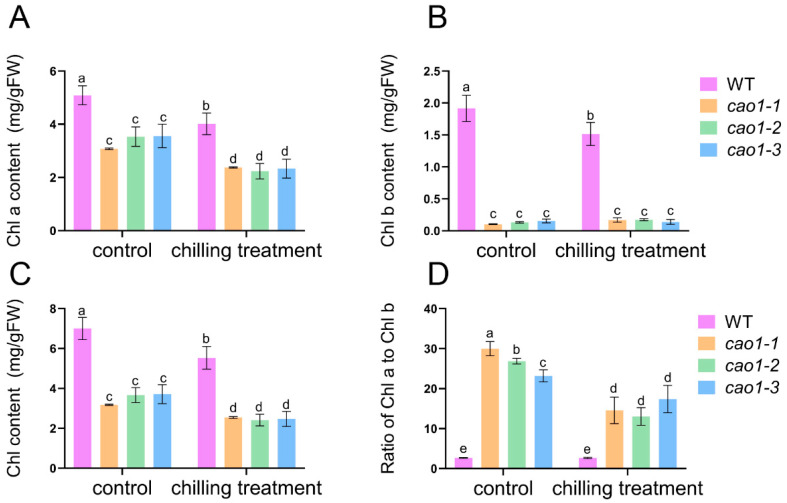
Analysis of chlorophyll metabolism in the WT and *OsCAO1* knockout mutants upon chilling stress. (**A**) Chl *a* content. (**B**) Chl *b* content. (**C**) Total chlorophyll content. (**D**) Ratio of Chl *a* to Chl *b*. Control: normal temperature; chilling treatment: low-temperature treatment. Data are means ± SD (*n* = 3). Different letters indicate significant differences among plant materials by two-way analysis of variance (temperature and plant materials) (*p* < 0.05).

The main function of *OsCAO1* is to catalyze the conversion of Chl *a* to Chl *b*, and chlorophyll level is also one of the key indicators for assessing plant cold tolerance [35]. To investigate the role of *OsCAO1* in chlorophyll metabolism under low-temperature stress, Chl *a*, Chl *b*, total Chl, and the ratio of Chl *a* to Chl *b* were measured in the mutants and WT seedlings under chilling stress. As shown in Figure 5, the total Chl, Chl *a*, and Chl *b* of the WT were significantly higher than those of the *OsCAO1* knockout mutants under normal temperature and low-temperature conditions. Chilling stress decreased Chl *a* in the WT by 20.57%, while the stress decreased Chl *a* in *cao1-1*, *cao1-2*, and *cao1-3* by 22.79%, 36.68%, and 34.45%, respectively (Figure 5A). Chl *b* in the WT decreased by 20.88% at low temperatures compared with normal temperatures; however, Chl *b* in the mutants remained at a very low level both at room temperature and low temperatures (Figure 5B). After low-temperature treatment, the total Chl of the WT and mutants decreased significantly. (Figure 5C). Chilling stress did not change the WT Chl *a*/Chl *b* ratio, while the stress decreased the Chl *a*/Chl *b* ratio in *cao1-1*, *cao1-2,* and *cao1-3* by 51.42%, 51.53%, and 24.94%, respectively (Figure 5D). The decrease in Chl *a* and the Chl *a*/*b* ratio in the *OsCAO1* knockout mutants caused by low temperatures were greater than that in the WT, indicating that the insufficient function of *OsCAO1* leads to the hypersensitivity of chlorophyll metabolism to low-temperature stress.

### 2.6. OsCAO1 Helps Maintain the Normal Morphology of Chloroplasts under Chilling Stress

**Figure 6:**
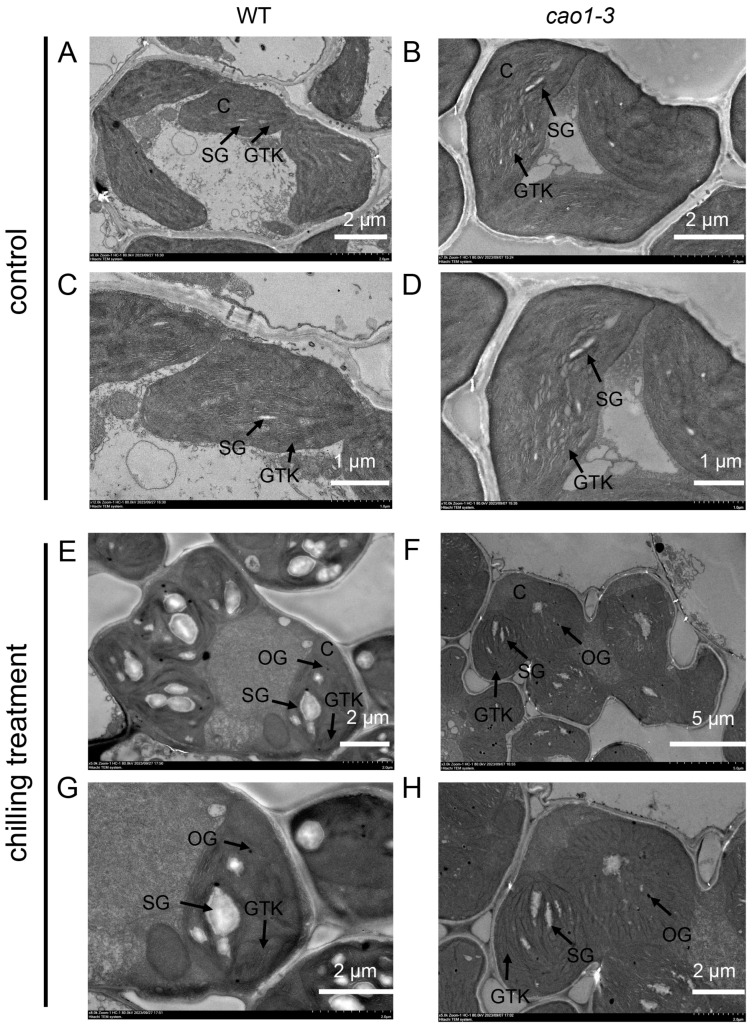
Transmission electron microscopy images of chloroplasts in the WT and *cao1-3* at the seedling stage. (**A**) WT under normal temperature. (**B**) *cao1-3* mutant under normal temperatures. (**C**) The magnified image of (**A**). (**D**) The magnified image of (**B**). (**E**) WT under low temperatures. (**F**) *cao1-3* mutant under low temperatures. (**G**) The magnified image of (**E**). (**H**) The magnified images of (**F**). C, chloroplast; SG, starch grain; GTK, grana thylakoid; OG, Osmiophilic granule.

In plants, chilling stress affects chloroplast development, causing abnormal morphology of the chloroplast [36]. To determine whether the cold hypersensitive phenotypes of *OsCAO1* knockout mutants were accompanied by chloroplast morphological changes, the ultrastructure of chloroplasts of the WT and *cao1-3* mutant grown under ambient and low-temperature conditions were observed by TEM (transmission electron microscopy). In seedlings grown under a normal temperature, the chloroplasts appear fully developed in the WT and *cao1-3* mutant, and the grana lamellae were arranged in an orderly manner and uniformly distributed, except that the thylakoid stacking of *cao1-3* mutant is not as tight as that in the WT (Figure 6A–D). A short period of chilling stress (12 °C for 2 d) did not significantly alter the grana thylakoid (GTK) morphology in the WT but caused a significant increase in starch granules (SG) (Figure 6E,G). However, under chilling stress, the GTK in the *cao1-3* mutant had a more disorderly arrangement in the chloroplast with indistinct thylakoid stacking (Figure 6E–H). These results suggested that the functional disruption of *OsCAO1* is averse to the maintenance of chloroplast morphology, especially GTK stacking, under low-temperature stress.

### 2.7. Knockout of OsCAO1 Results in Yield Loss

As reported previously, *OsCAO1* knockout significantly reduced the photosynthetic rate [22]. To explore whether the knockout of *OsCAO1* in our study affected yield, some main agronomic traits were determined. In comparison with the WT, the mutants had significantly less plant height, 1000 gain weight, seed setting rate, and grain yield per plant (Table 1). Compared with the wild type (123.00 ± 3.09 cm) for plant height (29.03 ± 0.66 g), thousand gain weight (84.58 ± 2.5%), and seed setting rate, the plant height, 1000 gain weight, and seed setting rate in the *OsCAO1* knockout mutants were reduced by 13.95–16.28%, 7.96–18.65%, and 19.15–21.75%, respectively (Table 1). However, the mutations did not change the tiller number, the primary panicle length, or the primary branch numbers (Table 1). It was indicated that *OsCAO1* deficiency not only leads to a decrease in cold tolerance in rice seedlings but also has a negative impact on yield.

### 2.8. OsCAO1 Affects the Chilling Response to Antioxidant Gene Expression

The enzyme proteins encoded by *OsCATB* and *OsAPX2* promote the clearance of excess ROS in cells, thereby enhancing the tolerance of rice to abiotic stress [37,38]. The expression levels of *OsCATB* and *OsAPX2* in the *OsCAO1* knockout mutant were higher than those in the WT at normal temperatures. However, after low-temperature treatment, the expression levels of these two genes in the mutant were significantly lower than those in the WT (Figure 5A,B).

OsMYB3R-2 protein is an R1R2R3 type MYB transcription factor, which can coordinate the cell cycle and the derived *DREB/CBF* pathway to improve the chilling stress response of rice [39]. It was shown that under normal temperatures, the expression levels of *OsMYB3R-2* were similar in different genetic materials. Low-temperature treatment significantly increased the expression of *OsMYB3R-2*, especially in the WT, where the expression level was significantly higher than in the mutants (Figure 5C). *OsMYB30* negatively regulates cold tolerance by inhibiting the expression of the *BMY* gene that controls maltose content [40]. *OsCAO1* did not affect the mRNA levels of *OsMYB30* at normal temperatures (Figure 5D). The expression of *OsMYB30* in both the WT and *OsCAO1* knockout mutants was up-regulated in response to low temperatures, while the up-regulation amplitudes of the mutants were significantly higher (Figure 5D). These results implied that the *OsCAO1* knockout mutants were chilling sensitive, possibly because the expression levels of the chilling tolerance-promoting genes, such as antioxidant genes, were lower, while the RNA levels of negative regulators, such as *OsMYB30,* were higher in the mutants upon chilling stress.

## 3. Discussion

CAO is a Rieske-type monooxygenase that converts Chl *a* into 7-hydroxymethyl Chl *a*, which is then further oxygenated to form Chl *b* in a two-step process [5,10]. The functions of the plant CAO family include responding to abiotic stresses and regulating resistance to adversity, in addition to catalyzing the synthesis of Chl *b*, which affects photosynthetic efficiency. In maize, the *ZmCAO1* gene mutation in the natural *yellow-green leaf* (*ygl*) mutant reduced the enzyme activity of CAO, resulting in increased ROS production and reduced waterlogging tolerance [41]. In rice, *OsCAO1*-deficient mutants exhibited increased levels of reactive oxygen species and poorer heat tolerance [20]. In this study, we first revealed that *OsCAO1* was a cold-responsive gene (Figure 2). Subsequently, we revealed for the first time the function of *OsCAO1* in promoting cold tolerance at the seedling stage in rice by constructing knockout mutants through gene editing technology for phenotypic analysis. (Figure 3C).

In our study, *OsCAO1* knockout mutants were not only significantly less cold tolerant but also had reduced yield. *OsCAO1* knockout mutants had a reduced seed setting rate and 1000 grain weight, but there were no significant differences in the tiller numbers compared with the WT (Table 1). The *pgl* mutant, generated by EMS mutagenesis in the background of a *japonica* rice variety Yunyin, harbors a single-base substitution in the coding region of *OsCAO1*, which results in a premature translation termination with only 318 aa (541 aa length in the WT) [20]. The tiller number and seed setting rate of the *pgl* plants were lower than those of the WT plants, but the 1000 grain weight was not changed by the mutation [20]. A near-isogenic line, *fgl* (*faded green leaf*), was generated through a process of backcrossing a leaf color mutant with ZF802 over a ten-generation period and had a two-base deletion in the ninth exon of *OsCAO1*, resulting in two amino acid substitutions (D522R, A523H) and only eighteen aa deletions in the C-terminus [21]. Compared with its recurrent parent, Zhefu 802 (ZF802, an early-season *indica* rice variety), *fgl* had significantly lower Chl *b* content, a higher 1000 grain weight and grain weight per plant, and a similar seed setting rate and No. of grains per panicle [21]. Our knockout mutants and *pgl* both showed a decrease in yield, although they had different down-regulated yield factors, whereas the yield of *fgl* increased (Table 1) [20,21]. This may be because the number of C-terminal amino acids missing in our mutants was similar to that in *pgl*, while the number of C-terminal amino acids missing in *fgl* is too small (both *cao1-1* and *cao1-3* lacked 269 amino acids, *cao1-2* lacked 273 amino acids, and *pgl* lacked 223 amino acids, whereas *fgl* lacked 18 amino acids at the C-terminal). All the above materials had reduced Chl *b* content, but their agronomic traits were not completely the same. Although all the mutations occurred in the C-domain, their effects were different. It is necessary to deeply dissect the specific roles of various amino acids at the C-domain in the catalytic activity and the regulation of yield and stress response in the future. Structural characterization of *OsCAO1* through an in silico approach similar to *AtCAO* in *Arabidopsis* and further conducting research on *OsCAO1’s* crystal structure and function is very valuable [42].

Overexpressed *OsCAO1* in ZF802 background showed elevated Chl *b*, together with increased tiller numbers, 1000 grain weight, and grain weight per plant, suggesting that the higher expression of *OsCAO1* was responsible for agronomic traits’ improvement [21]. Since the mutants we constructed under the background of 9311 showed a decrease in cold tolerance and yield (Figure 3, Table 1), we carefully speculate that the overexpression of *OsCAO1* may potentially enhance both cold tolerance and yield in rice varieties, such as the widely used restorer line 9311 for hybrid rice. As genetically modified crops are currently not allowed by policies in many countries and regions, future research needs to search for *cis*-acting elements in its promoter and upstream negative regulatory transcription factors to improve the expression level of *OsCAO1* in rice. By searching for elite alleles in natural germplasm and introducing the natural elite alleles, novel non-transgenic rice varieties with high *OsCAO1* expression could be created. As demonstrated by our study, there are various *cis*-elements in *OsCAO1*’s promoter, and its expression was closely related to the temperature and circadian clock (Figure 1 and Figure 2). As one of the most important genes involved in photosynthesis for photosynthetic organisms on Earth, the evolution of CAO genes in various species has adapted to temperature changes, sunlight, Earth rotation, and revolution cycles (the reasons for the evolution of circadian clock mechanisms) in their respective habitats. We hope to find *OsCAO1* elite alleles, which are more adaptable to adverse temperatures. Therefore, manipulating *OsCAO1* through the utilization of its elite alleles has the potential of increasing both rice yield and chilling tolerance simultaneously, which are often inversely related.

**Figure 7:**
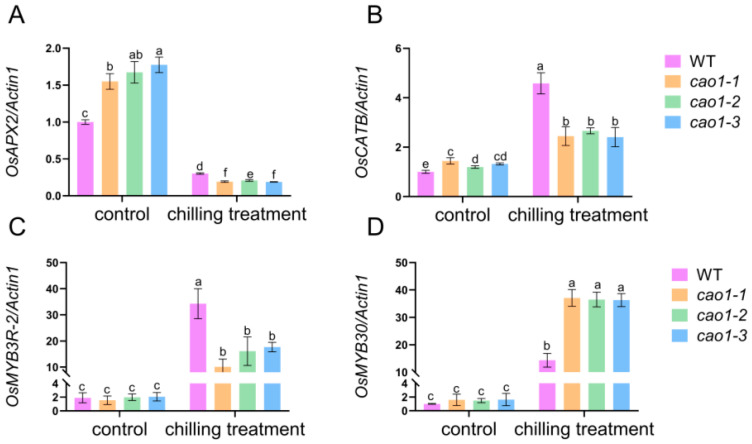
The regulation of *OsCAO1* on the expression profile of genes for antioxidant and chilling tolerance regulation. (**A**) *OsAPX2*. (**B**) *OsCATB*. (**C**) *OsMYB3R-2.* (**D**) *OsMYB30*. Data are means ± SD (*n* = 3). Different letters indicate significant differences among plant materials by two-way analysis of variance (temperature and plant materials) (*p* < 0.05). Chilling treatment: 12 °C for 24 h. For every RT-qPCR analysis, rice *OsActin1* was performed as a reference to detect its transcript level in various samples.

*OsCAO1* is exclusively localized and plays a pivotal catalytic role in the chloroplast [21]. Chloroplasts can sense low temperatures and are the first and most severely affected organelles partly by the production of excessive ROS in plants [43,44,45]. Excessive ROS and MDA are toxic to cells at low temperatures, leading to changes in cell membrane permeability and an increase in electrical conductivity [46]. To figure out the effect of *OsCAO1* in chloroplast response to chilling stress, we investigated the chloroplast morphology and ROS production in *OsCAO1* knockout mutants under chilling conditions. The ROS, MDA, and relative EL in the mutants were significantly higher than those in the WT (Figure 4). However, the activities of antioxidant enzymes (POD and SOD) for scavenging toxic ROS and MDA were significantly lower in the mutants than the WT (Figure 4). It was indicated that, in addition to maintaining normal chlorophyll metabolism at room and low temperatures (Figure 5), *OsCAO1* is helpful in suppressing the excessive ROS production in chloroplasts under low-temperature stress (Figure 4). Moreover, the inner structure of chloroplasts was less impaired with the function of *OsCAO1* in the WT (Figure 6). Then, *OsCAO1* may function in the chloroplast to transfer cold signals to the nucleus through plastid-to-nucleus retrograde signaling to optimize the gene expression of various cold-related genes, such as *OsCATB*, *OsAPX2*, *OsMYBS3R-2*, and *OsMYB30* (Figure 7) [37,38,39,40,47,48]. However, in-depth studies in the future are needed to provide essential evidence for the hypothesis.

In conclusion, our study uncovered the regulatory role of *OsCAO1* in chilling tolerance. However, the mechanism underlying how *OsCAO1* senses and transduces the cold signal downstream remains unknown. It is critical to identify the upstream transcription factors and the key motifs in the *OsCAO1* promoter that regulate *OsCAO1* gene expression in response to cold stimuli. In the future, the modification of cold-responsive motifs in the *OsCAO1* promoter region could be realized in two ways: (1) gene editing technology and (2) the utilization of elite haplotypes in natural germplasm resources. The modification of the *OsCAO1* promoter that confers an elevated expression of *OsCAO1* to achieve the optimal balance of cold tolerance and photosynthetic efficiency will help to cultivate cold-tolerant rice varieties with high yields.

## 4. Materials and Methods

### 4.1. Plant Material

The rice variety 9311 was used in this study. The gene-editing lines of *OsCAO1* (termed as *cao1* knockout mutants), *cao1-1*, *cao1-2,* and *cao1-3*, were generated through the CRISPR/Cas9 system (BioRun Co., Ltd., Wuhan, China) [49,50]. The identification of the gene editing region used in the primers is listed in Supplementary Table S1.

### 4.2. Chilling Treatment and Phenotypic Analysis

The mature seeds were surface sterilized after breaking dormancy. After soaking and germination, sprouted seeds were sown in seedling boxes containing 96-well plates. Seedlings were grown hydroponically with Kimura B nutrient solution under normal conditions (28 °C; 12 h light/12 h dark; 30,000 lux white light; 70% relative humidity) until the three-leaf stage. Seedlings were treated with low temperatures (12 °C) for 5 days and then transferred to normal conditions to recover for 7 days before counting the survival rate of the seedlings. The phenotypic analysis was performed as reported previously with minor modifications [51].

### 4.3. Promoter Sequence Analysis

A sequence of 2000 bp situated upstream of the *OsCAO1* gene (NCBI reference sequence NC_029265.1) was identified as the promoter sequence and sent to the PlantCARE database for *cis*-element predicting evaluation [31].

### 4.4. Measurement of Physiological Indexes

Seedlings were grown at normal temperatures (28 °C) for about 15 d, and after 2 d at low temperatures (12 °C), aerial leaves were collected for the determination of physiological indices. DAB (3, 3′-diaminobenzidine) staining and NBT (nitrotetrazolium blue chloride) staining were employed to quantify the content of superoxide anion radicals and hydrogen peroxide in plants. The NBT solution and DAB solution were prepared at a concentration of 1 mg/mL. The second leaf tissue, measuring 3 cm in length, was excised from the middle of the fully expanded portion. The DAB-stained samples were stained in the light for 4 days, while the NBT-stained samples were stained in the dark for 3 days and decolorized with 75% alcohol [52].

The determination of relative conductivity can be employed to ascertain the extent of damage to plant cell membranes. A quantity of 0.1 g of leaves was obtained and placed in 5 mL of distilled water. The leaves were then pressed with sterile absorbent cotton in order to ensure complete immersion in the water. After soaking at room temperature for 24 h, measure the conductivity R1 of the extraction solution using a conductivity meter (DSS-11A, Shanghai Chromatographic Instrument Co., Ltd., Shanghai, China). After heating the extraction solution in a boiling water bath for 30 min, the conductivity R2 was measured. The relative conductivity was calculated by R1/R2 × 100%. To quantify the content of MDA, 5 mL of 0.5% TBA (thiobarbituric acid) was added to the tissue fluid of rice leaves, and the water bath was maintained at 95 °C for 10 min. Following cooling in an ice bath, the supernatant was centrifuged, and the OD values were measured at 450 nm, 532 nm, and 600 nm. The methodology employed in determining relative conductivity and MDA content is based upon the previously utilized methodology, albeit with a few minor modifications [53].

Rice leaves were ground into a delicate powder using liquid nitrogen. Transfer 0.5 g of powder to a 15 mL centrifuge tube and immediately place it on ice. Add 5 mL of pH 7.8 phosphate buffer, shake, and mix well. Centrifuge at 4200 rpm for 20 min at 4 °C, transfer 1.5 mL of supernatant into a 2 mL centrifuge tube, and centrifuge at 13000 rpm for 10 min at 4 °C to obtain the rice tissue fluid. The relevant reagents were added in turn according to the product manual of the kits. The activity of SOD was quantified using the Micro Superoxide Dismutase (SOD) Assay Kit (Cat No. BC0175, Solarbio, Beijing, China). The POD activity was detected according to the manual of the Micro Peroxidase (POD) Assay Kit (Cat No. BC0095, Solarbio, Beijing, China).

### 4.5. Quantitative Real-Time PCR Analysis

To investigate whether the expression of *OsCAO1* responds to low temperatures, seedlings of wild type 9311 were cultivated at 28 °C in a 12 h light/12 h dark cycle. The seedlings at the three-leaf stage were transferred to low temperatures (12 °C) for 48 h, with samples collected every 4 h from ZT0 to ZT48 (Zeitgeber time, ZT).

To investigate the regulatory roles of *OsCAO1* on the expression of other known marker genes, the seedlings of the WT and *cao1* mutants after undergoing chilling treatment for 12 h were collected. The controls were seedlings grown at normal temperatures sampled at the same time points.

Rice seedling samples were quickly frozen in liquid nitrogen and ground into powder, which was later used for total RNA extraction (RNA easy isolation reagent, R701-01, Vazyme, Nanjing, China). The purity and concentration of the RNA samples were determined by an ultra-microspectrophotometer (K5600, Kaiao Technology Development Co., Ltd., Beijing, China) to ensure that the RNA samples had an A260/A280 between 1.8 and 2.0, an A260/A230 greater than 2.0, and a concentration of 400 ng/μL or more. A total of 1 μg of total RNA was used for reverse transcription to synthesize the first strand of cDNA (HiScript II qRT Super Mix Kit, Cat No. R223-01, Vazyme, Nanjing, China). The reverse transcription process used DNase I to remove genomic DNA, and the final double-stranded cDNA (after diluting 10 times) was used for real-time quantitative PCR. The PCR thermal cycle conditions were 95 °C for 5 min, 40 cycles at 95 °C for 10 s, and 60 °C for 30 s. The qPCR was conducted using a LightCycle ^®^ 480 System (Roche, Basel, Switzerland). The software to acquire raw data is LightCycler^®^ QC Test Software 3.0. The primers for the internal reference gene *OsActin* and the target genes for real-time quantitative PCR are shown in Supplementary Table S1 [40,54,55]. The above experiments were carried out in three independent biological replicates, and each biological replicate was with three technical replicates to analyze the expression level of the target genes by the 2^−ΔΔCt^ method [56].

### 4.6. Determination of Chlorophyll Content

The total chlorophyll (Chl) in the leaves was extracted with 95% ethanol [57]. The extract was analyzed with a spectrophotometer (HNSA-YQ-034). The contents of total Chl, Chl *a,* and Chl *b* were calculated from the absorbance values at 470, 645, and 663 nm.

### 4.7. Transmission Electron Microscopy (TEM)

The third leaves of the seedlings exposed to 12 °C for 5 d or grown continuously under 28 °C were sampled. Only the middle sections of the leaves without main veins were soaked in a fixed solution (2% (*v*/*v*) glutaraldehyde and 3% paraformaldehyde and phosphate buffer solution (PBS, pH 7.2) overnight at 4 °C and were then washed three times with PBS. The samples were post-fixed in 1% osmium for 2 h before washing three times with PBS at room temperature. After dehydration, the samples were embedded in Epon 812 (SPI Supplies, West Chester, PA, USA). Samples were sectioned and stained as previously described with minor modifications [58]. All sections were then stained and imaged by transmission electron microscopy (HT7800, Hitachi TEM System, Tokyo, Japan) at 80 kV.

### 4.8. Analysis of Yield-Related Traits

The yield-related traits (plant height, seed setting rate, 1000 grain weight, seed setting rate, panicle length, tiller number, number of primary branches, and grain yield per plant) of the WT and *OsCAO1* knockout mutants were analyzed. At least 10 independent plants of each line were measured.

### 4.9. Statistical Analysis

All trials were conducted in three biological repeats. The results are shown as mean ± SD and were analyzed statistically using SPSS (version 2.5). Different letters indicate significant differences among plant materials by two-way analysis of variance. Graphs were generated using GraphPad Prism (version 8.01). 

## Figures and Tables

**Table 1:** Agronomic traits of *OsCAO1* knockout mutants.

Traits	WT	*cao1-1*	*cao1-2*	*cao1-3*
Plant height (cm)	123.00 ± 3.09 ^a^	102.97 ± 3.80 ^b^	103.85 ± 4.73 ^b^	105.83 ± 6.55 ^b^
1000 grain weight (g)	29.30 ± 0.66 ^a^	23.8 ± 0.25 ^b^	26.96 ± 0.15 ^bc^	26.93 ± 0.38 ^bc^
Seed setting rate (%)	84.58 ± 2.5 ^a^	76.2 ± 1.7 ^b^	73.5 ± 8.2 ^b^	67.3 ± 6.6 ^c^
Panicle length (cm)	23.10 ± 1.07 ^a^	23.44 ± 1.82 ^a^	22.45 ± 1.15 ^a^	23.53 ± 1.42 ^a^
Tiller number	8 ± 1.58 ^a^	8 ± 1.93 ^a^	8 ± 1.5 ^a^	8 ± 1.13 ^a^
Primary branch number	12.9 ± 0.88 ^a^	12 ± 0.94 ^a^	12.2 ± 0.79 ^a^	12.8 ± 1.23 ^a^
Grain yield per plant (g)	31.98 ± 4.48 ^a^	22.63 ± 4.87 ^b^	22.38 ± 4.36 ^b^	26.94 ± 5.63 ^c^

The different superscripted letters of the trait indicate significant differences among those materials. Data are means ± SD (*n* = 10; *p* < 0.05, Student’s *t*-test).

## Data Availability

The data presented in this study are available in this published article.

## Supplementary Materials

The following supporting information can be downloaded at https://www.mdpi.com/article/10.3390/genes15060721/s1, Supplemental Table S1: Primers used in this study.
