# Supplementary material for: Knockout of the Chlorophyll a Oxygenase Gene OsCAO1 Reduces Chilling Tolerance in Rice Seedlings
Source: Genes (Basel). 2024 Jun 2;15(6):721. doi: 10.3390/genes15060721 (PMC11202714; doi:10.3390/genes15060721)
Supplement: Supplementary file 1 [file genes-15-00721-s001.zip › Supplemental table.pdf]

Supplemental Table S1 Primers used in this study

Primer name	Primer sequence (5’-3’)	PCR product length	Purpose
CAO1GE check F	ACGAGGTCAGAGTTAGCAGT	601bp	Identification of mutants
CAO1GE check R	TCTTGAAATGCACAGGACAGTT		
OsMYB3R-2 qF	CAGGGTTTCTATCTCGTTCC	139bp	quantitative Real-time PCR
OsMYB3R-2 qR	ATTTCCAAGCCCTTCCAC		
OsMYB30 qF	ACTCCGGGATGGAGATGAG	107bp	
OsMYB30 qR	GATGAACAGCTTGAGCCAGA		
OsCATB qF	GTTCGGTTCTCCACAGTCGT	311bp	
OsCATB qR	CCCTCCATGTGCCTGTAGTT		
OsAPX2 qF	CGTCTTCCTGATGCCACACA	116bp	
OsAPX2 qR	CATCTTCCCAGGGTGTGACC		
OsActin qF	GACCTTCAACACCCCTGCTA	90bp	
OsActin qR	GAGTCCAACACAATACCTGTGG		
OsCAO1 qF	ACACCTTCATCTGGGCTTCAAGGA	146bp	
OsCAO1 qR	AGATGCGTCGAACATTGCTTGGTG		
